# Supplementary material for: Rats Lacking Dopamine Transporter Display Increased Vulnerability and Aberrant Autonomic Response to Acute Stress
Source: Biomolecules. 2020 May 31;10(6):842. doi: 10.3390/biom10060842 (PMC7356162; doi:10.3390/biom10060842)

Supplementary Figure 1

A

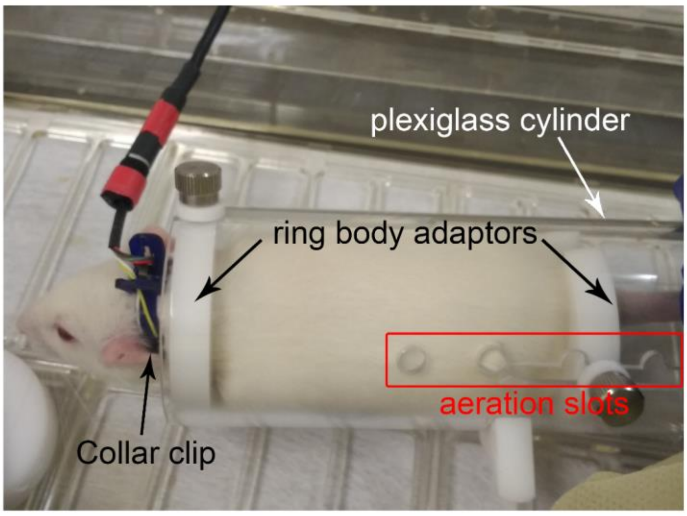

B

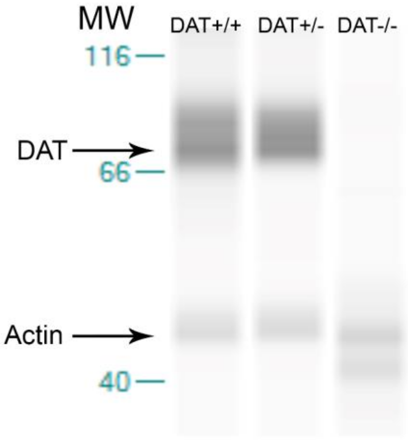

C

	DAT+/+	DAT+/-	DAT-/-
DAT	240771	153250	0
β-Actin	230956	225523	255066
Ratio DAT/β-Actin	1.0424973	0.6795316	0

D

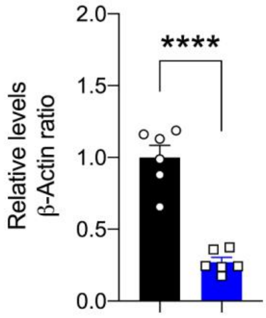

E

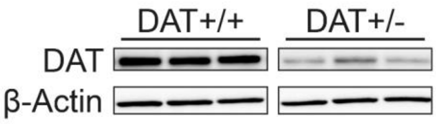

Supplementary Figure 2

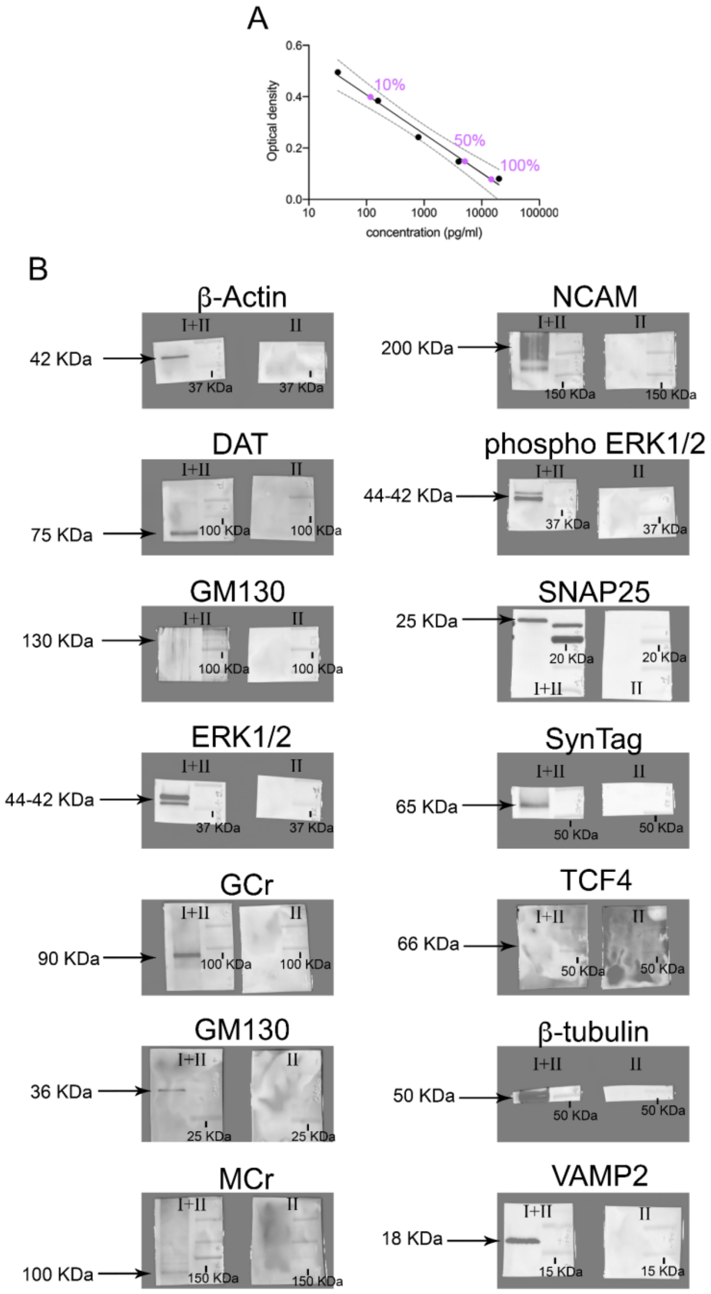

Supplementary Figure 3

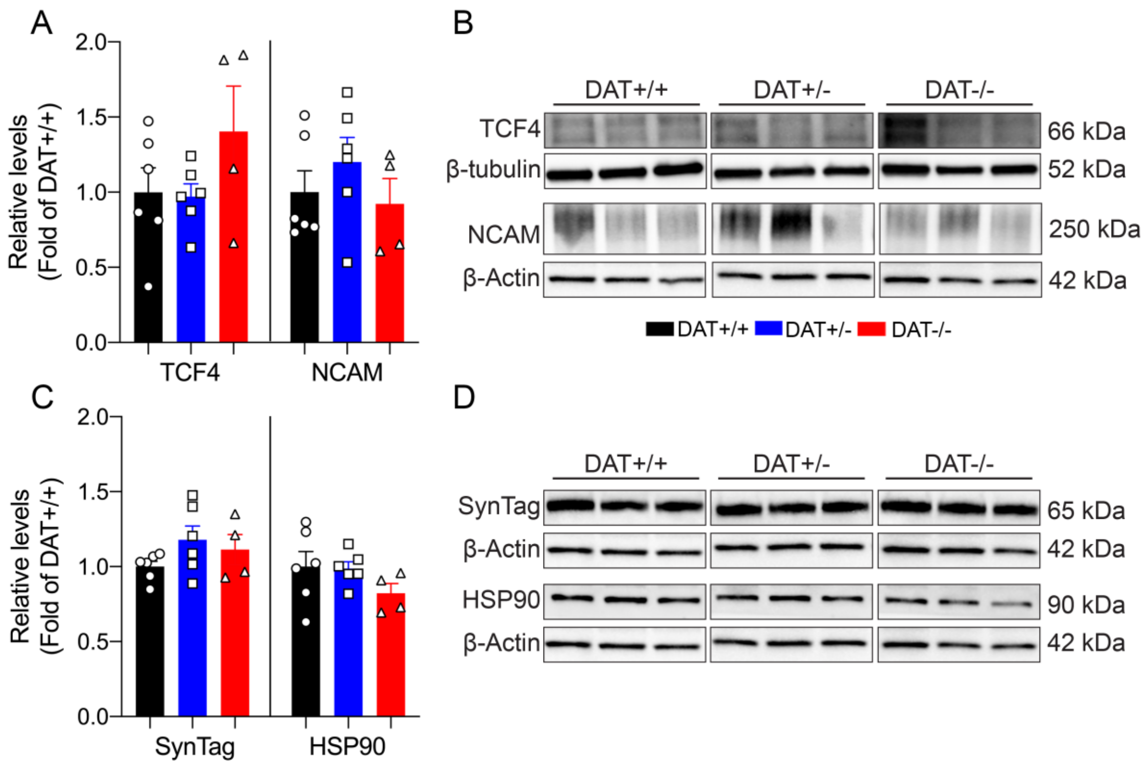

Supplement: Supplementary file 1 [file biomolecules-10-00842-s001.pdf]
